# Effects of Drag-Reducing Polymers on Hemodynamics and Whole Blood–Endothelial Interactions in 3D-Printed Vascular Topologies

**DOI:** 10.1021/acsami.3c17099

**Published:** 2024-03-15

**Authors:** Louis S. Paone, Matthew Szkolnicki, Brandon J. DeOre, Kiet A. Tran, Noah Goldman, Allison M. Andrews, Servio H. Ramirez, Peter A. Galie

**Affiliations:** † Department of Biomedical Engineering, Rowan University, Glassboro, New Jersey 08028, United States; ‡ Department of Pathology, Immunology, & Laboratory Medicine, College of Medicine, University of Florida, Gainesville, Florida 32611, United States

**Keywords:** bioprinting, hemodynamics, blood-enothelial interactions

## Abstract

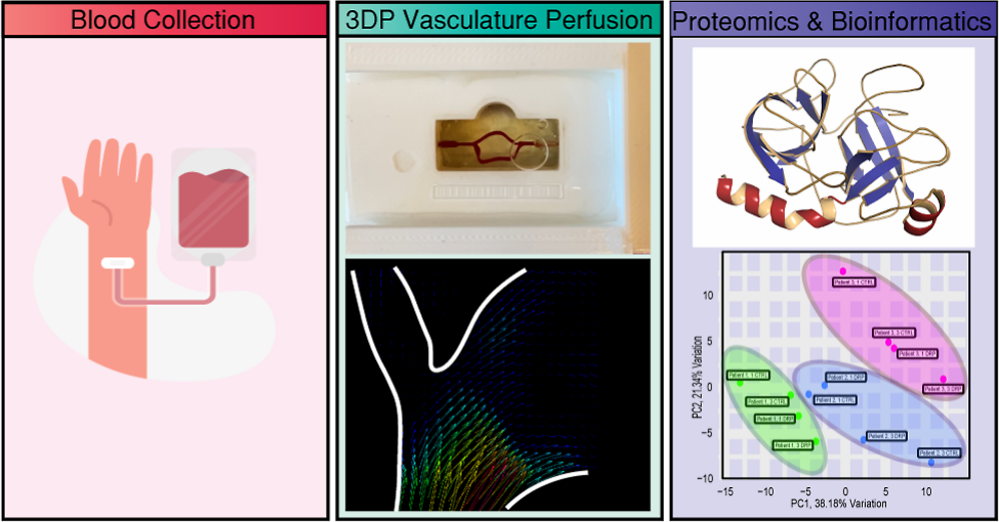

Most in vitro models use culture medium to apply fluid shear stress to endothelial cells, which does not capture the interaction between blood and endothelial cells. Here, we describe a new system to characterize whole blood flow through a 3D-printed, endothelialized vascular topology that induces flow separation at a bifurcation. Drag-reducing polymers, which have been previously studied as a potential therapy to reduce the pressure drop across the vascular bed, are evaluated for their effect on mitigating the disturbed flow. Polymer concentrations of 1000 ppm prevented recirculation and disturbed flow at the wall. Proteomic analysis of plasma collected from whole blood recirculated through the vascularized channel with and without drag-reducing polymers provides insight into the effects of flow regimes on levels of proteins indicative of the endothelial–blood interaction. The results indicate that blood flow alters proteins associated with coagulation, inflammation, and other processes. Overall, these proof-of-concept experiments demonstrate the importance of using whole blood flow to study the endothelial response to perfusion.

## Introduction

Hemodynamics have been studied in vitro since the 1930s when Fahraeus used glass capillaries to determine the effect of diameter on hematocrit.^1^ Since Fahraeus, in vitro flow studies with whole blood have provided insight into a diverse array of physiological processes, including coagulation and cell streaming. Yet the geometries used to study blood flow have not progressed much further beyond the glass capillaries used by Fahraeus. Researchers have used machining techniques to fabricate cylindrical geometries for hemodynamic studies, although these strategies lack vascular complexity.^2^ Soft lithography has been used to create microchannels with micron-scale resolution and network branching within PDMS-based substrates to measure red blood cell dynamics.^3^ Moreover, whole blood flow has also been applied to endothelial cells lining PDMS channels.^4^ However, the rigid walls of these channels and the rectangular cross sections caused by casting from molds are unable to mimic the cylindrical topologies of in vivo vasculature.^5^ Removing sacrificial materials from hydrogels polymerized within microfabricated chambers provides a better approximation of the circular cross-section of vasculature and the deformability of the vascular wall albeit with a limited capacity to recreate branched networks. For example, this method has been used to measure hemodynamics in channels within a collagen hydrogel using microparticle image velocimetry, though only in a straight geometry.^6^ Overall, previously described in vitro systems are unable to measure whole blood flow dynamics in a deformable, branched network topology.

**Table 1:** 

species	flow rate (mL min^–1^)	laser pulse width (μs)
bovine/human	1	2000
bovine	2	2000
bovine/human	3	1000
human	0.25	3000

The advent of digital light processing (DLP) provides a new avenue to study hemodynamics in topologies that are more faithful to in vivo vasculature, including interpenetrating and interconnected networks. 3D printing has frequently been used to approximate in vivo vasculature, though primarily using rigid substrates.^7,8^ DLP uses computer aided design in tandem with dichroic mirrors to project a filtered light source and facilitate step growth photochemical polymerization creating complex microvasculature within hydrogels.^9^ These printed hydrogel networks create complex flow regimes that can be studied in real time. The linearly elastic hydrogels deform during application of flow, mimicking the radial stretch in the vasculature. Currently, no laboratory has used this method to study hemodynamics. A recent advance in bioinks compatible with DLP allows the incorporation of spatially controlled, covalently bound peptides to the lining of the vessel.^10^ Whereas most bioinks compatible with DLP, such as GelMA and PEGDA, rely on nonspecific cell adhesion strategies, the material presented here provides a high degree of tunability using peptide motifs that mediate cell-ECM interactions.^11−13^ Additionally, utilizing multiarm polymeric subunits at high molecular weights yields faster printing kinetics^14,15^ leading to shorter exposure times that make this process robust and scalable. Taken together, this technology provides our laboratory with highly specific biomimetic modalities to study complex hemodynamics.

One flow phenomenon frequently interrogated in previous in vitro hemodynamic studies is the effect of drag-reducing polymers (DRPs) on blood flow. DRPs include high molecular weight, synthetic, hydrophilic polymers that reduce the effective fluidic resistance of blood flow through channels.^16^ Previous work has revealed a threshold of polymer molecular weight: polymers under 1 MDa fail to exhibit drag-reducing properties.^17^ In vitro studies suggest that DRPs attenuate the cell free layer at the boundary layer of blood flow,^18^ causing plasma skimming and subsequent redistribution of red blood cells within the microvascular network.^19^ In vivo, DRP addition to the blood has an inhibitory effect on human breast cancer cell metastasis,^20^ ameliorates hepatic injury after liver ischemia-reperfusion,^21^ and exerts a similar effect following myocardial infarction.^22,23^ Furthermore, DRPs have been shown to deplete localized amyloid-beta plaques along the vascular wall by increasing the perfused oxygen supply.^24^ Poly ethylene glycol derivatives are hypothesized to exert a drag-reducing effect due to their high degree of chain flexibility,^18^ but there remains uncertainty regarding the mechanisms by which DRPs reduce the pressure drop in whole blood flow. Moreover, in vitro studies have yet to investigate how the endothelial lining alters the effect of DRPs on hemodynamics. Here, the implementation of whole blood flow in complex vascular topologies lined with endothelial cells provides insight into how DRP-altered hemodynamics affect blood–endothelial interaction and may reduce pressure drop during pathologies associated with ischemia-reperfusion.

## Materials and Methods

### Bioprinting Vasculature

3D printed vasculature was fabricated using a LumenX + digital light processing printer (CellInk). A bifurcation topology was modeled using computer aided design (CAD, SolidWorks) iterated with computational fluid dynamics (CFD, COMSOL) to determine a geometry capable of inducing disturbed flow in one of the daughter branches at flow rates between 1 and 3 mL min^–1^. The CAD file was uploaded to the bioprinter in.stl format. For acellular experiments, poly ethylene glycol diacrylate (PEGDA) photoink was purchased along with PEGDA200 (CellInk). These inks were mixed at a ratio of 20:80% by volume to 0.8 mL and applied to a P100 PDMS-lined dish for photopolymerization. For experiments with cells lining the surface of the channels, poly ethylene glycol norbornene (PEG-Nor) hydrogels were printed. Briefly, lyophilized 20 kDa, 8-arm PEG-Nor polymer (JenKem Technologies) was reconstituted in phosphate-buffered saline (PBS). After solubilization, lithium phenyl-2,4,6-trimethylbenzoylphosphinate (LAP, Allevi) was added to poly ethylene glycol dithiol cross-linker (PEG-Dithiol, JenKem Technologies), and a photoabsorber, tartrazine (Millipore Sigma). Fabricated gels were washed in PBS 2× for 30 min following printing to remove unpolymerized polymer and excess reagent.

### Human and Bovine Whole Blood Collection and Preparation with Drag-Reducing Polymers

Bovine whole blood flow was used for the acellular experiments due to its availability and reduced safety requirements compared to human samples. However, human whole blood was needed for experiments in channels lined with human endothelial cells to eliminate any compatibility issues with human cells and bovine blood. For the acellular experiments, 500 mL of bovine whole blood was collected on ice with 10 mM sodium citrate following sacrifice at Bringhurst Meats in Berlin, NJ prior to transport to the Glassboro campus. For the cellular experiments, human whole blood was collected from volunteers by a mobile phlebotomy unit in Glassboro. Both bovine and human samples were prepared as 2 mL aliquots and equilibrated to room temperature. Four MDa poly ethylene oxide (PEO) was purchased from Millipore Sigma and solubilized at 10,000 ppm in PBS at 4 °C for 24 h. Several different concentrations of PEO were prepared in each whole blood sample for testing, including 10, 100, and 1,000 ppm. In all samples, fluorescently labeled Nile Red polystyrene particles (Spherotech) were added to measure velocity profiles by using microparticle image velocimetry.

### Branched Vessel Perfusion and Microparticle Image Velocimetry (μPIV)

PEGDA gels (for bovine whole blood perfusion) and PEG-Nor gels (for human whole blood perfusion) were perfused with a Genie Touch syringe pump for μPIV measurements. Gels were ported inside a perfusion chamber using 20-gauge PTFE catheter tips connected to Luer locks with tygon tubing (McMaster Carr). A 5 mL syringe was connected to inlet tubing in a temperature-controlled environment under a Nikon A1 epifluorescent microscope tandem μPIV laser (Dantec Dynamics). Bovine blood samples were subjected to 1, 2, and 3 mL min^–1^ flow rates, while human blood samples were measured at 0.25, 1, and 3 mL min^–1^ under the μPIV. To collect μPIV data accurately, a laser pulse width (defined as the time between firing of the dual lasers of the μPIV system) was input in the software to track separation of fluorescent beads (Table 1). Velocity data are shown in contour plots with colors corresponding to velocity magnitude and uniform length arrows indicating direction.

### Measurement of Hydrogel Mechanical Properties

A DHR-3 rheometer from TA Instruments was fitted with a 20 mm flat plate geometry. PEGDA and PEG-Nor cylinder geometries of height 4 mm and radius 2 mm were printed, washed in PBS, and kept overnight at 4 °C to replicate the workflow used when applying flow. Additionally, PEG-Nor cylinders were placed in EGM-2 and incubated at 37 °C/5% CO_2_ for 3 days to replicate the workflow of endothelial-seeded gels. Elastic modulus was measured through axial compression of the gels including 50% total strain at a rate set to 10% per second of the initial height. The modulus was equated to the slope of the stress-strain curve at approximately 10% strain magnitude. Representative images were taken at 1 image per second to calculate Poisson’s ratio. Elastic moduli and Poisson’s ratio were incorporated into the fluid-structure interaction module of the COMSOL model for both materials (Figure S1).

### Viscometry Testing of Whole Blood

Human and bovine whole blood viscosity were measured as a function of PEO concentration. A DHR-3 rheometer from TA Instruments was fitted with a 20 mm cone geometry for testing. PEO concentrations of 0, 10, 100, 500, and 1000 ppm were added to blood samples and tested in triplicate. A gap of 175 μM with a blood volume of 175 μL was added to the test platform prior to initiating a parametric sweep from 100 to 1000 s^–1^ strain rate to measure the resultant viscosity as a function of drag-reducing polymer concentration.

### Computational Modeling

Computational modeling was performed using COMSOL Multiphysics, a commercially available finite element software. The program solved for both the non-Newtonian fluid flow field and the deformation of the surrounding hydrogel. To do so, a time-independent fluid–structure interaction model was applied with a parametric sweep for several flow rates applied to the device. Using rheology data, the Carreau–Yasuda models of shear thinning fluids were calculated and implemented into the model for both bovine and human blood. A Navier–Stokes equation incorporating the momentum terms was used due to the Reynolds number being greater than 10 in some regions of the vessel. The elastic modulus and Poisson’s ratio of the PEGDA (Figure S1), and as previously reported, PEG-Nor,^10^ was used to predict deformation during whole blood flow perfusion using a linearly isotropic Hooke’s law constitutive equation. The geometry of the bifurcation topology and the surrounding hydrogel were both discretized with tetrahedral elements. Along the walls of the bifurcation topology, two layers of boundary elements were included in the mesh with a stretching factor of 1.2 along with a smoothing transition to the interior mesh. The boundary layer elements and three-dimensional views of the mesh are provided in Figure S2. Roller boundary constraints were used for the hydrogel to account for the slippage between the hydrogel and the surrounding holder. As mentioned previously, the fluid-structure interaction boundary conditions were set at the interface of the bifurcation topology and the surrounding hydrogel. The input boundary condition was set to the volumetric flow rate, and an outlet boundary condition of zero gauge pressure was used. A no-slip boundary condition was used along the walls of the bifurcation topology. The model predictions of velocity were reported using cut planes and cut lines through the geometry at the height corresponding to the focal plane of the microscope for each flow rate and PEO concentration. Contour plots were created at these planes based on the magnitude of the velocity, and uniform length arrows were added to these contours to indicate the direction of the flow within the topology. Cut lines were used to output the velocity magnitude as well as the wall deformation for comparison with experimental measurements. The COMSOL model file is publicly available for use by other laboratories conducting similar work.

### Boundary Layer Measurements in Straight Channels

300 μm-diameter cylindrical channels were created inside PDMS by inserting in a 30-gauge needle prior to polymerization. The needles were removed after the PDMS solidified, creating a cylindrical channel. The channels were perfused with blood with different concentrations of drag-reducing polymers by using a Genie Touch syringe pump. The syringe pump was equipped with a 5 mL syringe filled with 1 mL of bovine whole blood containing fluorescent tracers. The syringe pump was set at a constant flow of 600 μL/min to ensure a consistent Reynolds number of approximately 15, to match the maximum Reynolds number around the area of the bifurcation. A catheter tube, fitted with a catheter tip, was used to connect the syringe to the inlet of the PDMS devices. To collect μPIV data accurately, a laser pulse width was optimized with the software to ensure a clear separation of fluorescent beads. This visual inspection required that, between pulses, an individual bead should be displaced 1–2 segments of the particle’s size between pulses.

### Functionalizing PEG-Nor Vasculature with HAVDI Peptides

PEG-Nor gels were treated with HAVDI peptides (HAVDIGGGC, Genscript), a protein derived from *N*-cadherein, to facilitate hCMEC/D3 attachment and spreading within the bifurcation. HAVDI peptide was solubilized in acetonitrile at pH 8.0 at 50 mM and diluted 1:10 in 0.05 wt % Irgacure 2959. 50 μL of the 5 mM peptide solution was perfused into the lumen of the bifurcation gel and exposed to 10 mW/cm^2^ UV for 1 min. This process was repeated after inverting the gel to ensure full coverage of the peptide onto the surface of the channel. Gels were then washed in PBS 2× for 30 min to remove any unbound peptide.

### Endothelial Cell Seeding

P23 hCMEC/D3 endothelial cells were cultured until 90% confluent, trypsinized, and suspended at a concentration of 12.5 M/mL in EGM-2. 12 μL of 12.5 M/mL hCMEC/D3 were added into the lumen of the vessel every hour for 3 h incubated at 37 °C/5% CO_2_ in a six well plate with 1 mL of EGM-2. The cell seeding technique included 180° rotations every hour after reseeding for a total incubation of 4 h^25^ to allow cells to attach to the HAVDI peptide. Following, gels were kept in static culture for 3 days in EGM-2.

### Human Whole Blood Recirculation

Three male patients between the ages of 25–40 volunteered to donate 25 mL of blood collected into citrate-coated tubes (DHHS Federal Wide Assurance Identifier: FWA00007111). 3D-printed endothelialized vessels were fabricated as described above. Whole blood from each patient was collected and aliquoted for four different conditions for two different flow rates, 1 and 3 mL min^–1^, and two different concentrations of DRP (0 and 1000 ppm). Recirculating blood flow was perfused using a peristaltic pump controlled by an Arduino board. The lines of the fluidic circuit were primed including the dampener upstream of the perfusion chamber that housed the endothelialized bifurcated vessel. Whole blood flow from each patient was recirculated through the vessel for 30 min at 37 °C with ∼300 μL siphoned off from the circuit. Blood was then centrifuged for 15 min at 4 °C at 2000*g* with plasma then taken off the top and frozen at −80 °C.

### Bioinformatics

Raw data was obtained from the CHOP proteomics Core Facility via data independent acquisition. The data was expressed in counts above the detection limit from the mass spectrometer. All bioinformatics data was generated using Rstudio. 508 total proteins were detected across all samples and used for processing. Data underwent log base 2 transformation and median centering across all detected proteins within each condition. Following, three of the flow conditions, 1 mL min^–1^ DRP, 3 mL min^–1^ DRP, and 3 mL min^–1^ CTRL, were normalized to each patient’s 1 mL min^–1^ CTRL condition. Finally, all three patients were averaged across all three conditions (1 mL min^–1^ DRP, 3 mL min^–1^ DRP, and 3 mL min^–1^ CTRL). A principal component analysis was performed across all 508 detected proteins against each patient’s flow condition to justify normalization to each patient’s 1 mL min^–1^ CTRL condition. Each of the proteins was screened in comparison to the 1 mL min^–1^ control to determine up/down regulation. Finally, proteins were grouped depending on their functionality in the following categories: coagulation, inflammation, cell–matrix interactions, and reduction/oxidation. Then, *Z*-scores were calculated and reported in a heatmap per protein across all four conditions using the following equation
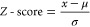
where *x* is the sample statistic, μ is the mean across all four conditions, and σ is the standard deviation across all four conditions to create tables for each category.

### Statistics

ANOVA and Tukey posthoc tests were done to determine the significant difference between viscosity and wall slip differences as a function of PEO concentration in blood. A *p*-value of 0.05 was considered significant. All studies presented include a sample number of at least 3.

## Results

### Validation of Vascular Geometry Using Computational Fluid Dynamics and MicroPIV

**Figure 1:**
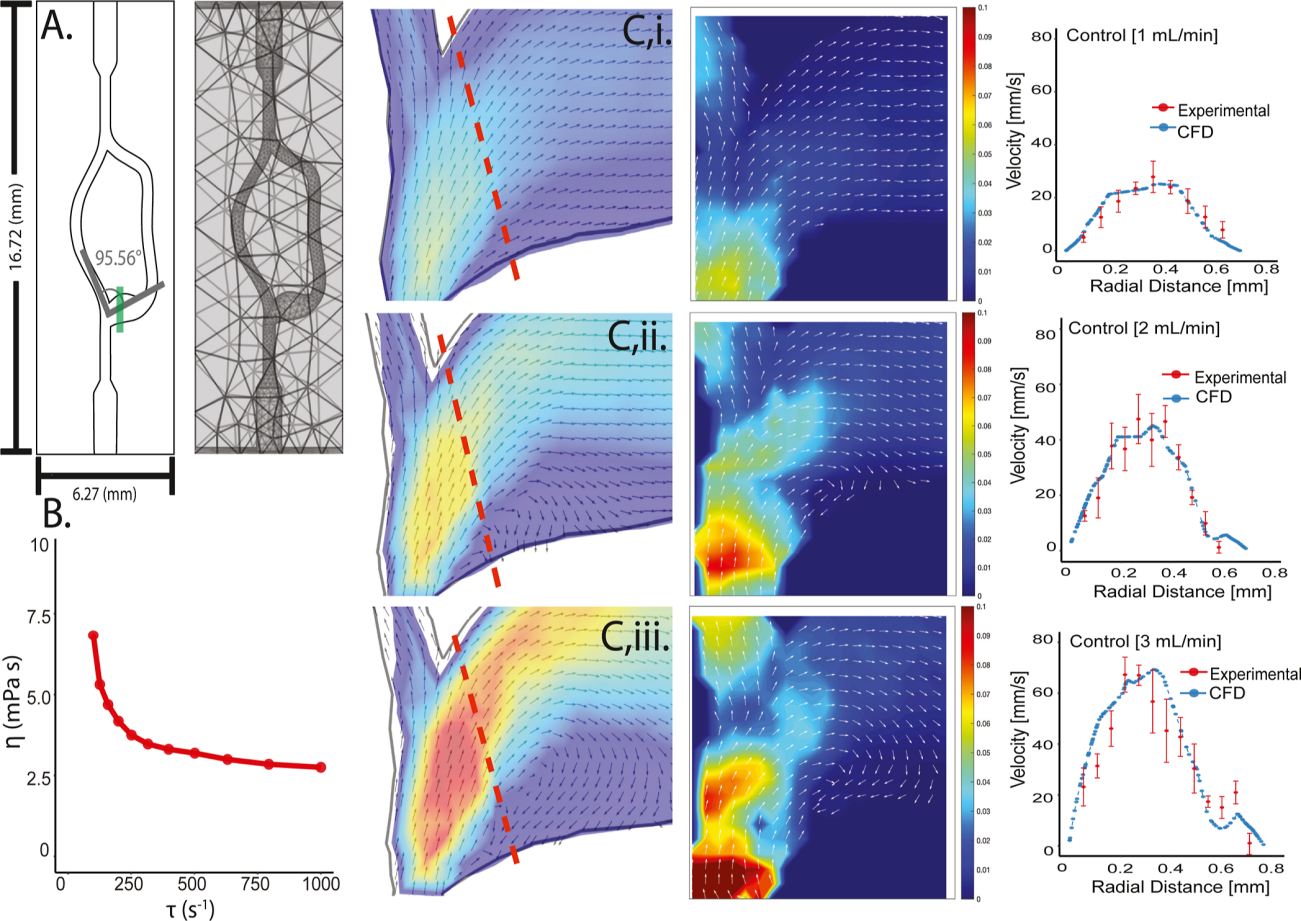
(A) Schematic of the 3D printed vascular model including the angle of the bifurcation, location of the μPIV sample collection zone, and the COMSOL mesh. (B) Viscometry of bovine whole blood control sample demonstrating non-Newtonian behavior. (C)(i–iii) From left to right, the COMSOL model, μPIV experimental vector map, and particle velocity were plotted across radial distance at 1, 2, and 3 mL min^–1^ all using bovine whole blood without DRPs (red dotted line = location of velocity plots).

A computational model was constructed to predict the velocity profiles of bovine whole blood perfused through an acellular 3D bifurcation model. The topology of the bifurcation was chosen to ensure flow separation at physiological flow rates. Flow separation is defined by the presence of a stagnation point along the wall and disruption of a parabolic boundary layer velocity profile. Figure 1A shows the discretization of the topology in commercial finite element software (COMSOL). Boundary layer elements were used to resolve the velocity gradient near the wall. The model incorporated a fluid-structure interaction algorithm to account for the deformation of the channel under flow. The bulk elastic modulus and Poisson’s ratio of the PEGDA-based photoink were measured using axial mechanical testing and input into the model (Figure S1). Moreover, shear viscometry (Figure 1B) was used to measure the shear thinning properties of whole bovine whole blood. Figure 1C compares simulated velocity profiles along with those measured by using microparticle image velocimetry (μPIV) at specific levels in the channel height. The velocity profiles of the predicted and measured values demonstrated substantial overlap along a radially oriented line (red dotted line in the predicted flow field). These results validate the accuracy of the computational model, and indicate that flow separation occurs above a flow rate of 2 mL min^–1^ at the bifurcation.

### Drag-Reducing Polymers Negate Flow Separation

**Figure 2:**
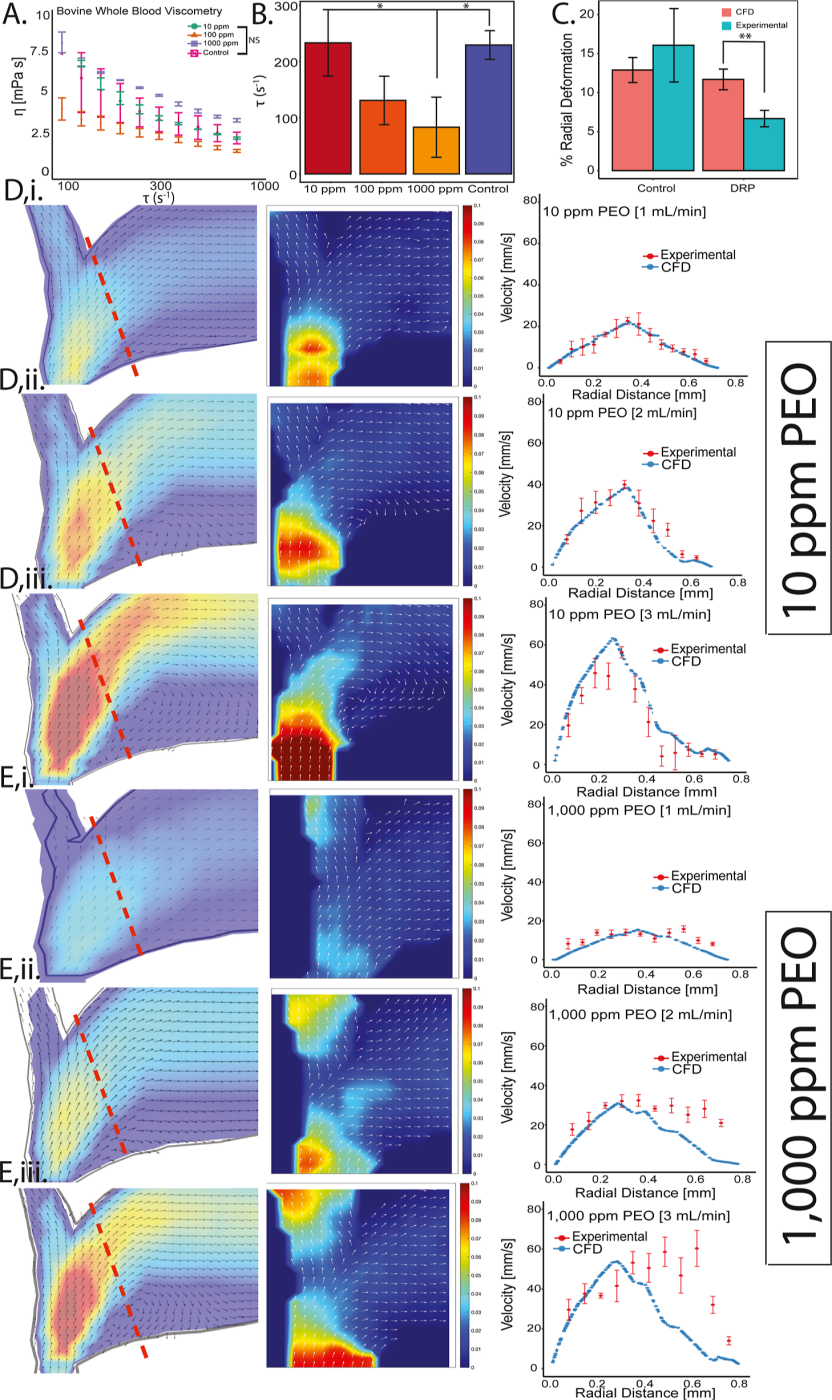
(A) Viscometry of titrated PEO in bovine whole blood in comparison to the control. (B) Shear rate as a function of PEO concentration in a straight channel perfused with 0.6 mL min^–1^ bovine whole blood. (C) Percent expansion of the bifurcation bicep with and without DRPs comparing experimental and computational results. (D)(i–iii) 10 ppm of PEO added to bovine whole blood perfused at 1, 2, and 3 mL min^–1^. (E)(i–iii) 1000 ppm PEO (1000 ppm) added to bovine whole blood perfused at 1, 2, and 3 mL min^–1^ (red dotted line = location of velocity plots). *(D,E) From left to right, COMSOL model, μPIV experimental vector map, and particle velocity plotted across radial distance at 1, 2, and 3 mL min^–1^(* = *p* < 0.05, ** = *p* < 0.005).

Previous in vitro and in vivo studies have demonstrated that the addition of long polymer chains to blood reduces flow separation at different scales, and also mitigates the pressure drop across the vascular bed.^16,18,26^ Therefore, several concentrations of poly(ethylene oxide) (PEO) were added to the blood to determine their effect on the flow field of whole blood within the bifurcation topology. Figure 2A shows viscometry testing of the blood, indicating that higher concentrations substantially alter the viscosity of the blood. Flow through cylindrical 300 μm-diameter PDMS channels also indicates that the PEO concentration significantly affects the shear rate of whole blood perfused at the same flow rate at the PDMS wall (Figure 2B). At low concentrations, however, e.g., 0.01 mg/mL, the PEO does not significantly affect the blood viscosity or the shear rate at the wall of the cylindrical channel. The computational model was altered to account for the effect of the polymers on blood viscometry. Given the effect of drag-reducing polymers on the pressure drop across the vasculature, the percent change in the radius of the vessel near the bifurcation was measured in both the computational model and the μPIV experiments. Figure 2C indicates close agreement in the control condition but a significant difference in radial deformation from computation and experiment in the DRP condition. The inability of the computational model to accurately predict blood flow containing DRPs, even when accounting for its effect on blood viscosity, was also apparent in the flow velocity measurements. At the lowest PEO concentration tested (10 ppm), the model and the measured velocity values show considerable overlap, as shown along a radially oriented line at the bifurcation (red dotted line in Figure 2D) for each of the three flow rates tested. However, at the highest PEO concentration (1000 ppm), the model and the measured values show less agreement. The discrepancy is most apparent on the near wall of the bifurcation, where the model predicts flow separation and a large recirculation zone that is not observed in the experimental measurements (Figure 2E). Previous studies have hypothesized that the effect of the drag-reducing polymers is caused by changes in red blood cell trafficking, which cannot be modeled using the continuum approach of the finite element model. Specifically, studies suggest that the presence of long chain polymers alters collision dynamics of red blood cells within a flow field.^27^ Nonetheless, these results indicate that addition of high concentrations of the drag-reducing polymer mitigates flow separation of whole blood through the bifurcation topology.

### Incorporation of Endothelial Cells in the Bifurcation Model

**Figure 3:**
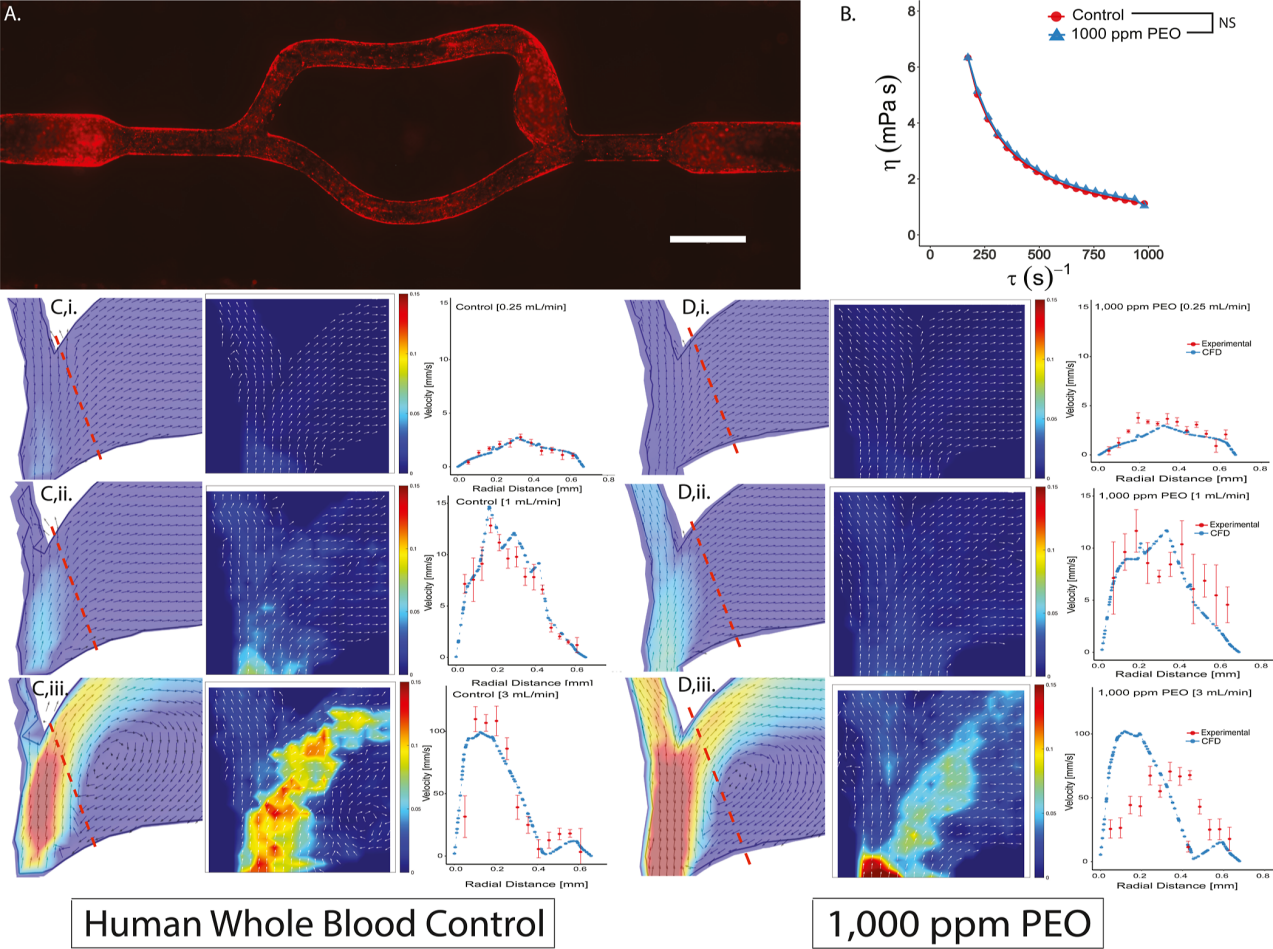
(A) 4× stitched epifluorescent microscope scan of transfected RFP P23 hCMEC/d3 cell line seeded on HAVDI peptide and cultured for 3 days. (B) Viscometry of human whole blood control and 1000 ppm of PEO. (C,D) Characterization of hemodynamic profiles of human whole blood in the presence of an endothelium both with/without PEO. *(C,D) From left to right, the COMSOL model, μPIV experimental vector map, and particle velocity plotted across radial distance at 0.5, 1, and 3 mL min^–1^. (scale bar = 1 mm).

Since the effect of the drag-reducing polymer occurs mostly near the boundary layer of the whole blood flow, experiments were conducted to determine whether endothelial cells lining the bifurcation affect the mitigation of flow separation in whole blood containing PEO. In order to incorporate endothelial cells into the model, a different photoink formulation was required. Specifically, a PEG-Norbornene bioink was devised to facilitate the binding of HAVDI peptides to the surface of the bifurcation topology to facilitate cell adhesion. This method, described previously by our laboratory,^10^ provides more control over cell–matrix adhesion than nonspecific cell adhesion strategies like incorporating gelatin methacrylate into the bioink. The elastic properties of PEG-norbornene were input into the computational model to accurately predict the vessel deformation. These simulations assumed that the presence of the endothelium did not substantially alter the mechanical properties of the PEG-norbornene hydrogels. Figure 3A shows an image of fluorescently labeled endothelial cells lining the entire length of the 3D-printed bifurcation. Bovine blood could not be used for these studies due to potential interactions between human endothelial cells and bovine blood cells. Therefore, human blood was used for this set of experiments, and additional viscometry was conducted to determine its shear thinning properties with and without drag-reducing polymers (Figure 3B). As the figure indicates, the addition of the polymers did not significantly affect the viscosity of human whole blood over the range of shear rates tested. Similar to the results in acellular channels with bovine blood, the control condition showed strong agreement between the predicted and measured velocity profiles. At 3 mL min^–1^, there is a clearly defined recirculation zone due to flow separation near the wall of the bifurcation (Figure 3C). However, addition of 1000 ppm PEO again results in a discrepancy between the predicted flow profile and the measured velocities. Similar to the bovine whole blood experiments in the acellular channels, a recirculation region is predicted, but the measured profiles showed no flow separation, and the velocity profiles indicated differences at the near wall of the bifurcation (Figure 3D). Overall, these results indicate that the presence of endothelial cells lining the channel does not interfere with the effect of drag-reducing polymers on flow separation at the bifurcation.

### Effect of Flow Regimes on Proteomic Profile of Whole Blood

**Figure 4:**
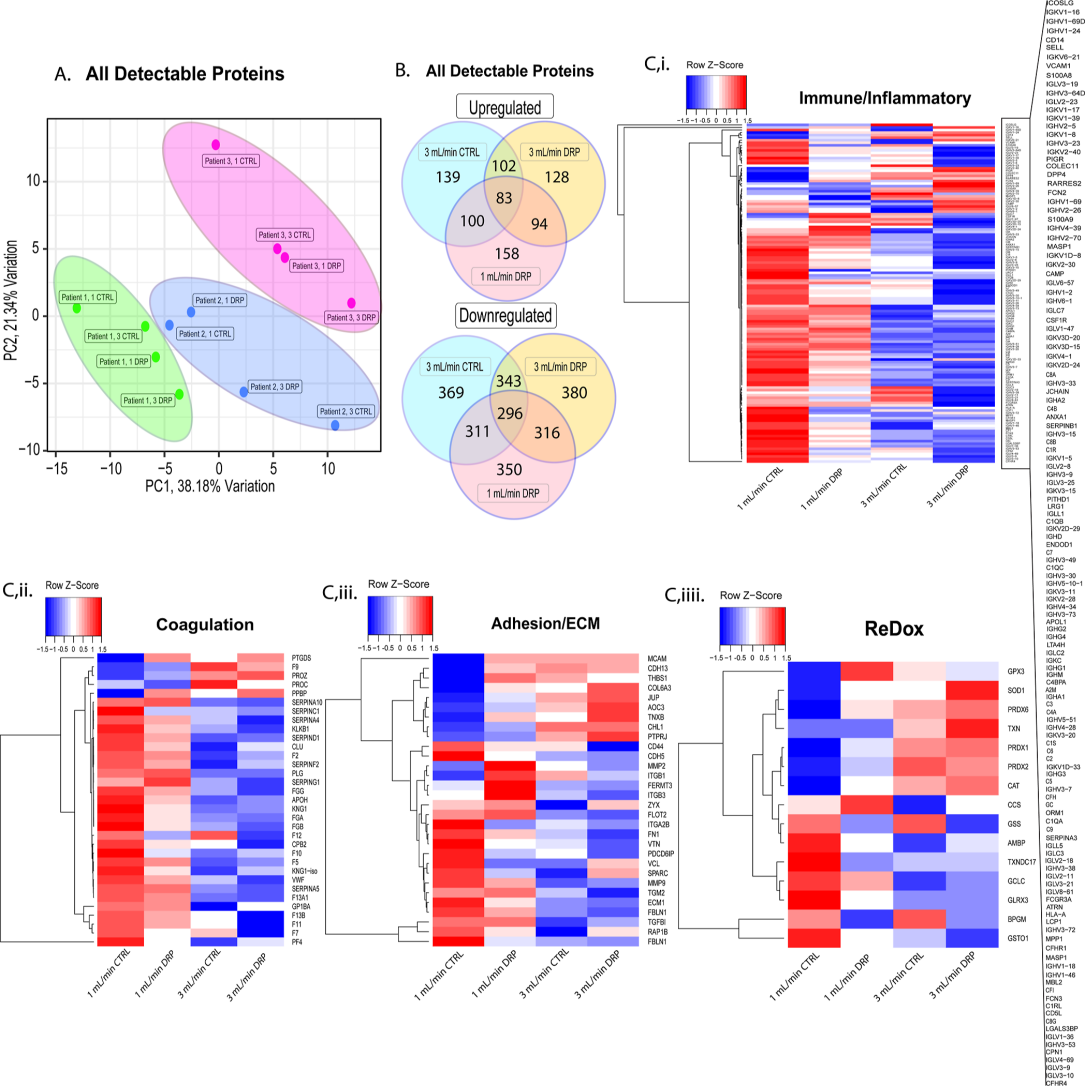
(A) Principal component analysis of all flow conditions run across the three patients tested. (B) Analysis of upregulated and downregulated proteins and the agreement between each of the flow conditions tested averaged across all patients. (C) *Z*-score heatmaps per protein across all conditions for categorized proteins based on physiological functionality.

Having demonstrated that drag-reducing polymers mitigate recirculation of whole blood at the 3 mL min^–1^ flow rate, experiments were conducted to determine whether the difference in flow profiles resulted in changes in the levels of proteins associated with inflammation, coagulation, extracellular matrix interactions, and redox markers expressed by the endothelium. The flow conditions of 1 and 3 mL min^–1^, described in Figure 3, were repeated to represent laminar and recirculatory flow respectively to induce disturbed flow, which we have previously shown adversely affects endothelial tight junctions.^28^ Human whole blood recirculation was performed for 30 min in the 3D-printed endothelialized bifurcation model with the blood collected following each flow condition for proteomics screening. A principal component analysis (Figure 4A) was performed across all detectable proteins to better understand how patients were grouped across the flow conditions. The PC analysis indicated that the patient’s own baseline physiology superseded any changes captured relative to flow rate or with the addition of DRPs, as evidenced by strong grouping among patient samples. Based upon this conclusion, the data needed to be manipulated accordingly to appropriately compare between flow conditions. Relative expression levels of proteins were acquired through data independent acquisition analysis (DIA) transformed, centered, and normalized to the 1 mL min^–1^ control flow condition to average and compare levels across patients. After protein expression levels were averaged across each patient, each flow condition was reported as upregulated or downregulated in comparison to the control (Figure 4B). Overall, both the addition of DRPs and flow rate resulted in more downregulation than upregulation over the entire spectrum of detected proteins. *Z*-score heatmaps were then used to better understand differences with respect to increased flow rate and the addition of DRPs on inflammatory, coagulation, ECM, and redox-associated proteins (Figure 4C). With *Z*-scores calculated across each condition per protein, the increase in the flow rate yielded larger differences in expression levels regardless of whether flow separation was present.

## Conclusions

DLP 3D-printing provides an unprecedented opportunity to implement realistic vascular topologies in vitro, but there are several reasons why previous studies have not exploited this feature to interrogate hemodynamics and blood–endothelial interactions. First, photoinks commonly used to print complex, intravascular topologies rely on nonspecific endothelial interactions with polymers like gelatin methacrylate, impeding the formation of a robust and confluent endothelial monolayer lining the channels. However, our laboratory has recently developed a DLP-compatible photoink capable of spatial patterning of peptide binding motifs in the channel wall,^10^ which is used in this study to fully endothelialize the printed topology. Second, perfusion of whole blood in cell-seeded channels in vitro is complicated by the potential of coagulation, increased pressure drop due to the higher viscosity of blood compared to culture medium, and difficulty visualizing flow with fluorescent tracers. The methods used in this study overcome these issues by preventing contact between the blood and any metal surfaces, using a hydrogel with sufficient mechanical properties to sustain increased pressure without bursting or tearing, and increasing the density of fluorescent tracers used for microparticle image velocimetry (μPIV). This approach can evaluate hemodynamics in the printed channels; which is first assessed by μPIV using cow blood in acellular channels and then with human blood in cellularized channels.

The combination of μPIV with computational fluid dynamics, pairing experiment with simulation, helps to better characterize the fluid flow regimes within the 3D-printed topology. Similar to its impact on experimental work, whole blood also increases the complexity of computational analysis. For example, the results include viscometry measurements to assess the shear rate-dependent properties of the whole blood with and without the addition of drag-reducing polymers (DRPs), data that are then modeled by a Carreau formulation to account for these non-Newtonian effects. Additionally, the blood flow rate also affects the dilation of the channel due to the pressure drop, requiring the inclusion of a fluid-structure algorithm to track the deformation of the vessel wall during perfusion. Incorporating these tools into the computational model produces a strong agreement between the velocity vectors measured by μPIV and those generated by the CFD code. Nonetheless, the computational model is incapable of predicting the lack of flow separation in blood treated with DRPs. Several studies have interrogated the mechanisms underlying the effect of DRPs on blood flow,^1,16,29^ with a consensus that the polymers affect red blood cell streaming and trafficking.^3,30−32^ However, the continuum nature of the computational model is unable to capture these effects, and is, therefore, a limitation that future studies may improve.

In addition to assessing hemodynamics in this system, the model also probes the interactions between blood cells and the endothelium. Previous endothelialized in vitro models primarily use culture medium to mimic the mechanical (e.g., shear stress^33^) and biochemical (e.g., nutrient concentration and osmolarity^34^) stimulation exerted by blood flow on endothelial cells in vivo. Although perfusing culture medium is advantageous in some respects, e.g., shear stress is linearly correlated with shear rate for Newtonian flow, culture medium cannot capture the complexity of whole blood. Moreover, the effect of blood flow on the endothelium is not unidirectional: the mechanical and biochemical stimuli experienced by the endothelium lead to changes in gene and protein expression that in turn alter the behavior of blood cells being perfused through the vessel. This feedback loop cannot be captured by perfusing culture medium through endothelialized in vitro models. The proteomic analysis presented in this study suggests that differences in the fluid regimes, specifically volumetric flow rate and corresponding Reynolds number, exert a more substantial effect on the proteomic profiles present in plasma than the addition of drag-reducing polymers. Nonetheless, there are still limitations of this in vitro approach. First, the bifurcation topology does not mimic a specific region of the vasculature. Rather, the motivation for its design is to induce flow separation at flow rates corresponding to physiological Reynolds numbers. Moreover, the flow circuit is unable to capture the effects of the entire circulatory system, e.g. the factors secreted by the liver.^35^ Nonetheless, the model serves as a proof-of-concept to encourage future studies to interrogate how the interaction between blood and the endothelium regulates vascular function.

Despite its limitations, the model remains capable of providing some insight into the effects of DRPs on blood–endothelial interactions and invokes considerations for using these polymers therapeutically. The LC–MS proteomics data indicate that flow rate exerts a greater effect on the detection of certain groups of proteins in plasma, e.g., inflammation, coagulation, etc., than DRP concentration. The high flow rate conditions (3 mL min^–1^ control and 3 mL min^–1^ DRP) exhibit profiles more similar to those of 1 mL min^–1^ DRP and 3 mL min^–1^ DRP, despite the finding that DRPs mitigate flow separation. These findings suggest that for the 30 min that blood was perfused through the vessel in these experiments, the effect of higher flow rates on the blood supersedes the consequences of disturbed flow. Future studies can interrogate longer time points to determine whether prolonged periods of disturbed flow yield more substantial effects on blood chemistry. Moreover, given the effects of DRPs on the boundary layer and the importance of the endothelial glycocalyx, future studies can also determine the role of the glycocalyx in mediating blood–endothelial interactions, given its established role in endothelial mechanotransduction.^36−38^

## Data Availability

Raw and processed data files will be accessible in the Harvard Dataverse Repository at the following link: https://dataverse.harvard.edu/dataverse/galielab.
