# Supplementary material for: Effects of Drag-Reducing Polymers on Hemodynamics and Whole Blood–Endothelial Interactions in 3D-Printed Vascular Topologies
Source: ACS Appl Mater Interfaces. 2024 Mar 15;16(12):14457–66. doi: 10.1021/acsami.3c17099 (PMC10982934; doi:10.1021/acsami.3c17099)
Supplement: Supplementary file 1 — am3c17099_si_001.pdf [file am3c17099_si_001.pdf]

**Supplementary Materials**

# Supplementary Materials for

## The effects of drag-reducing polymers on hemodynamics and whole blood-endothelial interactions in 3D-printed vascular topologies

Louis Paone *et al.*

*Corresponding author. Email: galie@rowan.edu

**This PDF file includes:**

Figure S1

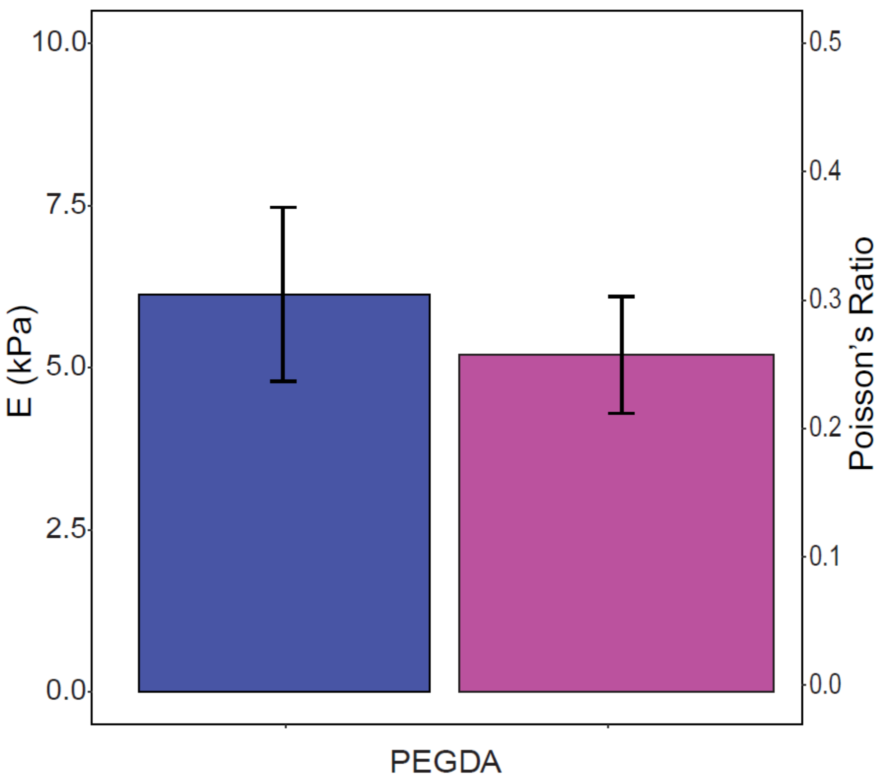


Supplemental Figure 1: Elastic modulus and Poisson's ratio of PEGDA measured via axial mechanical testing (N=3).

Figure S2

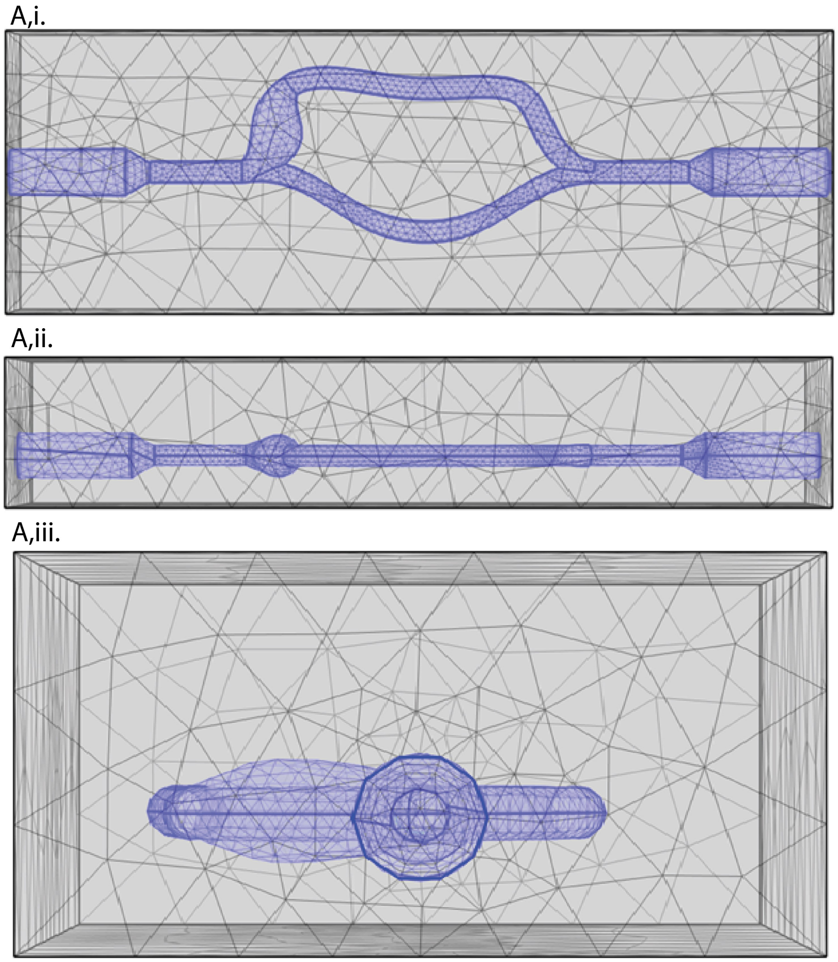


Supplemental Figure 2: Tetrahedral mesh used for the fluid-structure interaction finite element model of the bifurcation topology used in blood flow.
